# Marine Polysaccharides from Algae with Potential Biomedical Applications

**DOI:** 10.3390/md13052967

**Published:** 2015-05-15

**Authors:** Maria Filomena de Jesus Raposo, Alcina Maria Bernardo de Morais, Rui Manuel Santos Costa de Morais

**Affiliations:** CBQF—Centro de Biotecnologia e Química Fina, Laboratório Associado, Escola Superior de Biotecnologia, Universidade Católica Portuguesa/Porto, Rua Arquiteto Lobão Vital, Apartado 2511, 4202-401 Porto, Portugal; E-Mails: fraposo@porto.ucp.pt (M.F.J.R.); abmorais@porto.ucp.pt (A.M.B.M.)

**Keywords:** polysaccharides, algae, bioactive, biomedical, pharmaceuticals, therapeutics, drug delivery, regenerative medicine, wound management

## Abstract

There is a current tendency towards bioactive natural products with applications in various industries, such as pharmaceutical, biomedical, cosmetics and food. This has put some emphasis in research on marine organisms, including macroalgae and microalgae, among others. Polysaccharides with marine origin constitute one type of these biochemical compounds that have already proved to have several important properties, such as anticoagulant and/or antithrombotic, immunomodulatory ability, antitumor and cancer preventive, antilipidaemic and hypoglycaemic, antibiotics and anti-inflammatory and antioxidant, making them promising bioactive products and biomaterials with a wide range of applications. Their properties are mainly due to their structure and physicochemical characteristics, which depend on the organism they are produced by. In the biomedical field, the polysaccharides from algae can be used in controlled drug delivery, wound management, and regenerative medicine. This review will focus on the biomedical applications of marine polysaccharides from algae.

## 1. Introduction

Contemporary tendency for natural products to be applied in medicine and to promote health has put some emphasis in research on marine organisms, including macro- and microalgae, and cyanobacteria. Extensive literature on the health benefits and uses as food or as drug carriers of brown, red and green seaweeds, and the polysaccharides (**PS**) they produce, was published in the last decade [1,2,3,4,5,6,7,8]. However, comparatively, there are only a handful of research papers on microalgae [9,10,11,12,13,14], despite the richness of their composition and the ability to make them grow.

Polysaccharides already proved to have several important properties [3,8,12,15,16,17,18,19,20,21,22]. However, the attempts to establish a relationship between the structures of the **PS** and their bioactivities/actions have been a challenge due to the complexity of this type of polymers. In fact, aside from the homogalactan from *Gyrodinium impudicum* (a dinoflagellate) [23], the β-glucan from *Chlorella vulgaris* (a green microalga) [24] and the **PS** from a few species of seaweeds (Table 1, Table 2 and Table 3), most of these carbohydrates are highly branched heteropolymers with different substituents in the various carbons of their backbone and side-sugar components. Additionally, the monosaccharide composition and distribution within the molecule, and the glycosidic bonds between monosaccharides can be very heterogeneous, which is a real impairment for the study of their structures. Moreover, this heterogeneity also depends on the species, between strains of the same species, and on the time and place of harvest.

The **PS** produced by algae are presented in Table 1, Table 2 and Table 3, according to the group of macroalga, Phaeophytes, Rhodophytes, Chlorophytes, and in Table 4, which is relative to microalgae. Nevertheless, there are always some similarities between the **PS** from each group of seaweeds: often, fucoidans are extracted from brown algal species (Table 1), agaroids and carrageenans come from red macroalgae (Table 2), and ulvans are obtained from green seaweeds (Table 3). Regarding microalgae (Table 4), and as far as we know, there are not common names for their **PS**, to the exception of spirulan from *Arthrospira platensis*. There are species that, besides producing large amounts of these useful polymers, they secrete them out into the culture medium and these polymers are easily extracted [14].

**Table 1:** Marine species of brown macroalgae (**PHAEOPHYTES**) producing polysaccharides (**PS**): some structural features and applications.

Type of PS	Source	Structure		Action/Application	References
		Main Mono-Sugars/Disaccharide Units	Glycosidic Bonds of Backbone		
	**Chromophyta Dictyotales**				
Heterofucans S-fucans	*Canistrocarpus cervicornis* a.k.a. *Dictyota cervicornis*	Fuc		Anticoagulant, antioxidant; anti-proliferative	[2,25]
S-galactofucans	*D. menstrualis*	Gal, fuc, xyl, glcAc		Peripheral anti-nociceptive, anti-inflammatory, antioxidant; anticoagulant, anti-proliferative	[1,2,26]
	*D. mertensis*			antioxidant; anticoagulant, anti-proliferative	[2]
Heterofucans	*Dictyopteris delicatula*	Fuc		Anticoagulant, antioxidant, antitumor, anti-proliferative	[2,27]
	*D. polypodioides*	Fuc		Antitumor	[28]
S-galactofucans	*Lobophora variegata*	Gal, fuc		Antioxidant, anticoagulant, anti-inflammatory	[29,30]
Heterofucans	*Padina gymnospora*	GlcAc, fuc,	(1,3)- and (1,4)-β-d-glcAc	Antioxidant, anticoagulant, anti-thrombotic, antiviral	[2,31,32]
S-fucan	*P. tetrastromatica*	Fuc, gal, xyl, glcAc	(1,2)- and (1,3)-α-fuc		[33]
S-galactofucans; sPS; S-fucans	*Spatoglossum schröederi*	Gal, fuc, xyl; Fuc	(1,4)- and (1,3)-α-fuc	Anti-thrombotic; Peripheral anti-nociceptive; Anti-proliferative, anti-adhesive, antioxidant	[2,34,35,36,37,38]
	**Ectocarpales**				
S-galactofucans	*Adenocystis utricularis*	Gal, fuc, rham, uronic acid	(1,3)-α-fuc	Antiviral	[39]
S-fucans	*Cladosiphon okamuranus* a.k.a. *Okinawa mozuku*	Fuc, glc, glcAc	(1,3)-α-l-fuc	Anti-proliferative, antiviral, anti-inflammatory, antiadhesive, antitumor, immunomodulator; angiogenic, gastroprotective, cardioprotective, restenosis preventive	[15,22,40,41,42,43,44,45,46,47]
S-fucoidan	*C. novae-caledoniae*	Fuc		Antitumor	[48]
Fucans	*Leathesia difformis*	Fuc		Antiviral	[49]
LMW-S-fucans	*Nemacystus decipiens*	Fuc		Anticoagulant	[50]
	**Fucales**				
S-fucans; LMW-sPS; S-Laminaran; or otherwise modified	*Ascophyllum nodosum*	Fuc, xyl, gal, glcAc; Glc	(1,3)- and (1,4)-α-l-fuc (alternating); (1,3)- and (1,6)-β-glc	Immunomodulatory, anti-inflammatory, anticoagulant, anti-thrombotic, anti-metastatic, antitumor, antiadhesive, restenosis preventive; Anti-thrombotic, anticoagulant, angiogenic Antitumor, anticoagulant; serum hypocholesterolaemic, hypotensive, antibacterial, immunomodulator	[15,20,51,52,53,54,55,56,57,58,59,60,61]
S-fucans	*Fucus* spp. *F. vesiculosus*	Fuc, xyl, gal, glcAc	(1,3)- and (1,4)-α-l-fuc (alternating)	Immunostimulant, antiviral, antitumor, antiproliferative, antiadhesive, anticoagulant, antioxidant, anti-metastatic, anti-inflammatory; anti-angiogenic, antithrombotic (except *F. vesiculosus)*	[2,15,62,63,64,65,66,67,68,69,70]
Laminaran; S-laminaran or otherwise modified	*Fucus* sp.	Glc	(1,3)- and (1,6)-β-glc	Antitumor, decreases liver triglyceride, cholesterol and phospholipid levels; serum hypocholesterolaemic, hypotensive, antibacterial, immunomodulator anticoagulant	[56,59,61]
S-fucans	*Hizikia fusiforme* a.k.a. *Sargassum fusiforme*	Fuc, gal, man, glcAc	(1,2)-α-d-man alternating with (1,4)-β-d-glcAc; some (1,4)-β-d-gal	Anticoagulant, anti-thrombotic	[71,72]
Fucans	*Pelvetia fastigiata*	Fuc		Antiviral	[73]
LMW-S-fucans	*P. canaliculata*	Fuc		Antiviral	[74]
S-fucans	*Sargassum* spp.	Fuc, gal, xyl, uronic acid		Prevent hyperlipidaemia, normalize dislipidaemia	[75,76,77]
S-galactofucans	*Sargassum* sp.	Gal, fuc, rham, glcAc	(1,6)-β-d-gal and/or (1,2)-β-d-man	Antitumor	[28,62,78,79,80]
S-heterofucans	*S.filipendula*	Fuc		Antioxidant, anti-proliferative	[2,81]
S-fucoidan	*S. henslowianum*	Fuc		Anti-proliferative, antitumor	[75]
S-fucoidan	*S. horneri*	Fuc	(1,3)-α-l-fuc, (1,3)- and (1,4)-α-l-fuc	Antitumor, antiviral	[62,80]
LMW-fucoidan	*S. patens*	Fuc		Antiviral	[32]
sPS	*Turbinaria conoides*			Antioxidant	[82]
	**Laminariales**				
S-galactofucan	*Costaria costata*	Gal, fuc		Antitumor	[16]
S-fucans	*Ecklonia cava E. kurome*	Fuc, rham, gal, glcAc	(1,3)- or (1,6)-, and (1,4)-α-l-fuc	Anti-proliferative, antitumor, anticoagulant, antioxidant, antithrombotic, anti-inflammatory	[16,83,84,85,86,87,88]
Fucoidans; laminarans	*Eisenia bicyclis*	Fuc; Glc	(1,3)- and (1,6)-β-d-glc	Anti-proliferative, antitumor, anticoagulant; Antitumor	[83,89,90,91]
Laminaran; S-laminaran or otherwise modified	*Laminaria* sp (or *Saccharina*)	Glc	(1,3)- and (1,6)-β-glc	Antitumor, anticoagulant, decreases liver triglyceride, cholesterol and phospholipid levels; serum hypocholesterolaemic, hypotensive, antibacterial, immunomodulator	[56,59,61]
S-fucoidans	*Laminaria* spp.	Fuc, xyl, man, glcAc	(1,3)-α-l-fuc	Antioxidant, anticoagulant, antithrombotic, anti-adhesive, anti-proliferative, anti-inflammatory, anti-angiogenic, anti-metastatic	[15,52,83,92,93,94,95,96]
S-galactofucan	*L. japonica* a.k.a. *Saccharina japonica*	Gal, fuc	(1,3)- and (1,4)-α-l-fuc (alternating)	Anti-lipidaemic, increases HDL, antiviral, antitumor, immunomodulator, antioxidant neuroprotective	[3,15,97,98,99,100,101,102]
Fucoidans	*Lessonia vadosa*	Fuc		Anticoagulant	[103]
S-fucoidan	*Saccharina cichorioides* a.k.a. *Laminaria cichorioides*	Fuc		Antitumor, anticoagulant, anti-thrombotic	[104,105]
S-galactofucans fucoidan	*Undaria pinnatifida*	Gal, fuc, xyl, uronic acid	(1,3)- and (1,4)-α-l-fuc (alternating)	Antiviral, anticoagulant, antitumor, anti-proliferative, immunomodulatory, anti-inflammatory induced osteoblastic differentiation	[3,52,69,106,107,108,109,110,111]
LMW-S-fucans				Anticoagulant	[112]
Laminaran; S-laminaran or otherwise modified		Glc		Anticoagulant, antitumor; serum hypocholesterolaemic, hypotensive, antibacterial, immunomodulator	[56,59,61]

**Table 2:** Marine species of red macroalgae (**RHODOPHYTES**) producing **PS**: some structural features and applications.

Type of PS	Source	Structure		Action/ Application	References
		Main mono-Sugars/Disaccharide Units	Glycosidic Bonds of Backbone		
	**Rhodophyta Bangiales**				
S-galactan porphyran	*Porphyra* spp.	Gal	(1,3)-β-d-gal or (1,4)-α-l-gal	Antitumor, hypotensive, regulates blood cholesterol	[113,114]
sPS	*P. haitanensis*			Antioxidant	[115]
Porphyran	*P. yezoensis*			Antitumor, immunomodulatory, hypolipidaemic	[116,117,118,119]
	**Ceramiales**				
S-agarans	*Bostrychia montagnei*			Antiviral	[120]
S-agarans	*Cryptopleura ramosa*			Antiviral	[121]
	*Digenea simplex*			Antiviral	[122]
	**Corallinales**				
LMW-PS	*Corallina* sp.			Antiviral	[32]
	**Cryptonemiales**				
	*Cryptonemia crenulata*	Gal		Antiviral	[123]
S-agaran	*Gloiopeltis complanata*	Gal, Agal	[→3)-β-d-gal-(1→4)-3,6-α-l-Agal-(1→], and [→3)-β-d-gal-(1→4)-α-l-gal-(1→]		[114]
Agaroid-carrageenan	*G. furcata*	Gal	6-*O*-methyl-gal, 3,6Agal(1,3)-β-d-, and (1,4)-α-l-gal or (1,4)-α-l-Agal		[124]
	**Gelidiales**				
di-S-galactan	*Gelidium crinale*	Gal		Anticoagulant	[125]
S-agarans and hybrid dl-galactans	*Pterocladia capillacea*	Gal		Antiviral	[126]
					
	**Gigartinales**				
S-agarans S-galactans	*Aghardiella tenera*	Gal		Antiviral	[127,128]
S-λ-carrageenan	*Chondrus crispus*	Gal, Agal	(1,3)-α-d-gal, and (1,4)-β-3,6-Agal or (1,4)-β-d-gal (alternating)	Antiviral, anticoagulant, antithrombotic	[1,5,129,130,131]
LMW-sPS	*C. ocellatus*			Antitumor	[132]
S*-*galactans	*Euchema cottonii*	Gal		Antioxidant	[2]
S-κ-carrageenan	*E. spinosa*	Gal, Agal	(1,3)-α-d-gal, and (1,4)-β-3,6-Agal or (1,4)-β-d-gal (alternating)	Anticoagulant, anti-thrombotic	[5,130,131]
LMW-sPS	*Furcellaria lumbricalis*			Immunostimulant	[133]
S-galactans	*Gigartina acicularis*	Gal		Antioxidant	[2]
S-carrageenans	*G. skottsbergii*	Gal, Agal	(1,3)-α-d-gal, and (1,4)-β-3,6-Agal or (1,4)-β-d-gal (alternating)	Antiviral, anticoagulant	[130,131,134,135]
Hybrid dl-galactans	*Gymnogongrus torulosus*	Gal		Antiviral	[136]
LMW-PS	*Hypnea charoides*			Antiviral	[32]
LMW-S-carrageenans	*Kappaphycus striatus*	Gal, Agal	(1,3)-α-d-gal, and (1,4)-β-3,6-Agal or (1,4)-β-d-gal (alternating)	Antitumor, immunomodulator	[1,131]
S-λ-carrageenan	*Phyllophora brodiei*	Gal, Agal	(1,3)-α-d-gal, and (1,4)-β-3,6-Agal or (1,4)-β-d-gal (alternating)	Anticoagulant, antithrombotic	[130,131,137]
LMW-sPS	*Soliera chordalis*			Immunostimulant	[138]
S-carrageenans	*Stenogramme interrupta*	Gal, Agal	(1,3)-α-d-gal, and (1,4)-β-3,6-Agal or (1,4)-β-d-gal (alternating)	Antiviral	[130,131,139]
	**Gracilariales**			Antioxidant	[2]
sPS	*Gracilaria caudata*				
S-agarans S-galactans	*G.corticata*	Gal		Antiviral	[140]
sPS	*G. verrucosa*			Immunomodulator	[141]
	**Halymeniales**				
S-galactan	*Grateloupia indica*	Gal		Anticoagulant, antithrombotic	[137]
	**Nemaliales**				
S-mannans	*Nemalion helminthoides*	Man		Antiviral	[142]
Xylogalactans S-xylomannans	*Nothogenia fastigiata*	Xyl, gal Xyl, man		Antiviral, anticoagulant	[143,144,145]
	**Nematomatales**				
S-galactans	*Schizymenia dubyi*	Gal, uronic acid		Antiviral	[146]
S-λ-carrageenan	*S. pacifica*	Gal, Agal	(1,3)-α-d-gal, and (1,4)-β-3,6-Agal or (1,4)-β-d-gal (alternating)	Antiviral	[130,131,147]
S-galactan	*S. binderi*	Gal		Anticoagulant	[148]
	**Rhodymeniales**				
di-S-galactan; LMW-sPS	*Botryocladia occidentalis*	Gal		Anticoagulant; anti-venom	[149,150]
LMW-carrageenans	*Champia feldmannii*	Gal, Agal	(1,3)-α-d-gal, and (1,4)-β-3,6-Agal or (1,4)-β-d-gal (alternating)	Antitumor	[130,131,151]
	**Sebdeniales**				
S-xylomannans	*Sebdenia polydactyla*	Xyl, man		Antiviral	[152]

**Table 3:** Marine species of green macroalgae (**CHLOROPHYTES**) producing **PS**: some structural features and applications.

Type of PS	Source	Structure		Action/ Application	References
		Main Mono-Sugars/Disaccharide Units	Glycosidic Bonds of Backbone		
	**Chlorophyta Bryopsidales**				
sPS, including S-galactans	*Caulerpa* spp.			Antioxidant, anticoagulant, antithrombotic; antiviral, anti-proliferative, antitumor	[2,153,154]
sPS and derivatives	*C. cupressoides*	Gal, man, xyl		Anti-inflammatory, antinociceptive	[8,155,156]
LMW-PS sPS	*C. racemosa*	Gal, glc, ara, uronic acid		Antiviral; antitumor	[32,154,157]
S-arabinogalactans	*Codium* spp.	Gal, ara	(1,3)-β-d-gal	Anticoagulant, antithrombotic, antiviral	[124,153,158,159,160,161]
S-pyrulylated-galactans	*C. isthmocladum*		(1,3)-β-d-gal	Antioxidant, anticoagulant, anti-proliferative	[2,162]
	**Ulotrichales**				
S-mannans	*Capsosiphon fulvescens*	Man, glcAc, gal		Immunomodulator	[163]
S-rhamnans and LMW-S-rhamnans	*Monostroma latissimum*	Rham	(1,3)-α-l-rham, and (1,3)-α-l-rham or (1,2)-α-l-rham or (1→2,3)-α-l-rham	Antiviral, anticoagulant	[164,165,166,167,168]
S-rhamnans	*M. nitidum*	Rham, glc		Anticoagulant, antithrombotic, hepatoprotective, antitumor, immnunomodulator	[165,166,169,170,171]
	**Ulvales**				
Rhamnans	*Enteromorpha intestinalis*	Rham, xyl, glcAc		Antitumor, immunomodulator	[172,173]
LMW-sPS	*E. linza*			Anticoagulant	[174]
S-ulvans and derivatives	*E. prolifera*			Immunomodulator, antioxidant, hypolipidaemic	[124,175,176,177]
S-ulvans and derivatives	*Ulva* spp.	Rham, xyl, glc, glcAc, IduAc		Anti-adhesive, antiproliferative, hepatoprotective	[178,179]
sPS	*U. conglobata*	Rham, uronic acid		Anticoagulant	[180]
sPS	*U. fasciata*	rham		Antioxidant. antitumor	[181]
S-galactans sPS	*U. lactuca*	Rham, xyl, glcAc		Antioxidant, anti-proliferative, hypocholesterolaemic, hepatoprotective, antitumor; Antiviral, anti-inflammatory, antinociceptive	[90,182,183,184,185,186,187,188,189]
S-ulvans	*U. pertusa*	Rham, xyl, glcAc, iduAc	[→4)-β-d-GlcAc-(1,4)-α-l-rham3S-(1→], and [→4)-α-l-IduAc-(1,4)-α-l-rham3S-(1→]	Antioxidant, anti-proliferative, hypocholesterolaemic	[90,182,183,184,185]
LMW-S-ulvan or otherwise modified	*U. pertusa*			Antioxidant, hypotriglyceridaemic, decrease LDL- and increases HDL-cholesterol, immunostimulatory	[166,185,190,191]
S-PS	*U. rigida*	Rham, glcAc	β-d-glcAc-(1,4)-l-rham (disacharide)	Immunostimulatory	[178,192]

**Table 4:** Marine species of microalgae/blue-green algae producing **PS**; main neutral sugars.

Type of PS	Source	Main Neutral Sugars	Action/Application	References
	**MICROALGAE**			
	**Diatoms**			
sPS	*Cylindrotheca closterium*	xyl, glc, man, rham		[193,194]
sPS	*Navicula salinarum*	glc, xyl, gal, man		[193]
s-EPS	*Phaeodactylum tricornutum*	glc, man, xyl, rham	Anti-adhesive	[195,196,197]
EPS	*Haslea ostrearia*			[198]
EPS	*Nitzschia closterium*			[199]
EPS	*Skeletonema costatum*			
EPS	*Chaetoceros* spp.	rham, **fuc**, gal, man		[200]
EPS	*Amphora* sp.			[201]
	**Chlorophytes**			
sPS	*Chlorella stigmatophora*	glc, xyl, **fuc**,	Anti-inflammatory, immunomodulator	[195]
sPS	*C. autotrophica*			[202]
PS β-(1,3)-glucan	*C. vulgaris*	rham, gal, arab, 2-*O*-methyl-rham glc	Antitumor, infection preventive agent	[24,203,204]
EPS	*Dunaliella salina*	gal, glc, xyl, fru		[205]
EPS	*Ankistrodesmus angustus*			[201]
EPS	*Botryococcus braunii*	gal, **fuc**, glc, rham		[206,207]
	**Prasinophyte**			
sPS	*Tetraselmis* sp.		Anti-adhesive	[202]
	**Prymnesiophyte/haptophyte**			
sPS	*Isochrysis* sp.			[202]
	**Rhodophytes**			
sPS	*Porphyridium* sp.	xyl, gal, glc	Anti-inflammatory, immunomodulator, prevention of tumour cell growth, anti-adhesive, antiviral, biolubricant	[208,209,210,211,212,213]
sPS	*P. cruentum*	xyl, gal, glc, glcAc, 3-*O*-methyl-xyl	Antioxidant and free radical scavenging, immunomodulator, antiviral, antibacterial, antilipidaemic, antiglycaemic	[214,215,216,217,218,219,220,221,222]
sPS	*P. purpureum*		antiviral	[223]
sPS	*Rhodella reticulata*	xyl, rham, 3-*O*-methyl-rham, 4-*O*-methyl-gal	Antiviral, antilipidaemic, antiglycaemic, prevention of tumour cell growth	[208,213,219],
	*R. maculata*	xyl, gal, glc,3-*O*-methyl-xyl		[224,225]
	**Dinoflagellates**			
sPS	*Cochlodinium polykrikoides*	man, gal, glc	Antiviral	[226]
sPS	*Gyrodinium impudicum*	gal	Antiviral, anti-inflammatory, immunomodulator, anti-proliferative, prevention of tumour cell growth	[23,227,228,229]
	**CYANOBACTERIA**			
EPS	*Aphanothece halophytica*	glc, fuc, man, arab, glcAc		[230]
EPS s-Spirulan	*Arthrospira platensis*	gal, xyl, glc, fru rham, **fuc**, glc, 3-*O*-methyl-rham	Antiviral, antibacterial, prevention of tumour cell growth Anti-proliferative, anti-adhesive, anti-metastatic	[19,223,231,232,233,234,235]
sPS	*Anabaena*, *Gloethece*, *Nostoc Aphanocapsa*, *Phormidium*, *Synechocystis*, *Cyanothece*			[19]

Both micro- and macroalgae are excellent sources of **PS**, most of them being sulphated (**sPS**). They are associated with several biological activities and potential health benefits, making them interesting compounds for the application in pharmaceuticals, therapeutics, and regenerative medicine. Some of the beneficial bioactivities demonstrated by the crude **PS** and their derivatives, either *in vitro* or *in vivo*, upon various kinds of cell-lines and animal models, include anticoagulant and/or antithrombotic properties, immunomodulatory ability, antitumor and cancer preventive activity (as anti-proliferative agents, tumour suppressors or natural cell-killers). They are also good antidislipidaemic and hypoglycaemic agents, and can be powerful antioxidants, antibiotics and anti-inflammatory. For example, the sPS from *Enteromorpha* and *Porphyridium* have demonstrated strong antitumor and immunomodulating properties [173,211]; those from *Caulerpa cupressoides* and *Dyctiota menstrualis* are good antinociceptive agents [1,155], and the **sPS** from *Cladosiphon okaramanus* showed angiogenic, gastro- and cardioprotective bioactivities [15,46,47].

## 2. Some Structural Characteristics of Polysaccharides Produced by Marine Algae

The chemical structure of PS produced by macro- and microalgae may significantly determine their properties, namely physico-chemical and biochemical, and reflect their physical behavior and biological activities, as will be discussed further on in this review.

### 2.1. Macroalgae

Seaweeds (or marine macroalgae), whose **PS** have been studied more often, belong to the groups Chlorophyta (green seaweeds), Phaeophyceae (brown algae, Chromophyta) and Rhodophyta (red macroalgae).

Brown seaweeds usually contain fucoidans; the oligosaccharides obtained from the hydrolysis of fucoidans may often contain **gal**, **glc**, uronic acids, and/or other monosaccharides (Table 1), linked together and to the main chain by different types of glycosidic bonds. This is the case, for example, for the laminaran from *E. bicyclis* (Laminarales), or the galactofucan from *Sargassum* sp. (Fucales), and the fucan from *P. tetrastromatica* (Dictyotales) (Table 1). However, the structure complexity of these fucoidans makes difficult to establish a relationship between the **PS**-chains/composition and their biological actions, and/or some kind of protocols to design universal pharmaceuticals or other drug-like substances to prevent and/or cure specific diseases. This issue will be discussed later in this review.

The monosaccharide composition, the linkage types, the overall structure of fucoidans, and some of their di- and oligosaccharides were well explored by Ale *et al.* [75], Fedorov *et al.* [3] and Li *et al.* [103]. For example, Ale’s group [75] showed the difference between **sPS** from three species of *Fucus* by focusing on the various substituents at C-2 and C-4 carbons, despite the similarities of their backbones; they also highlighted the possible structures of fucoidans from two species of *Sargassum*, already suggested by Duarte *et al.* [78] and Li *et al.* [71]. Cumashi and coworkers suggested some structures for the backbone chain of several seaweeds [15]. Among them are the schemes for the components of the main chain showing either the (1,3)-, and (1,3)- and (1,4)-linked **fuc** residues or some di- and trisaccharide repeating units for *A. nodosum*, *C. okamuranus*, *L. saccharina* (a.k.a. *Saccharina latissima*), and some species of *Fucus*. On the other hand, Fedorov *et al.* [3] focused on the structures and bioactivities of different **sPS**, such as fucoidans (e.g., galactofucan from *Laminaria* (a.k.a. *Saccharina japonica*), and laminarans (e.g., the one from *E. bicyclis*) (Table 1).

Red macroalgae contain large amounts of **sPS** (Table 2), mostly galactans (agaroids and/or carrageenans), with alternating repeating units of 1,3-α-**gal** and 1,4-β-d-**gal** [236], and/or 3,6-anhydro**gal** (3,6-**Agal**) [237]. Substituents can be other monosaccharides (**man, xyl**), sulphate, methoxy and/or pyruvate groups, the pattern of sulphation dividing carrageenans into different families, for example, in C-4 for κ-carrageenan, and in C-2 for λ-carrageenan. In addition, the rotation of **gal** in 1,3-linked residues divides agaroids (l-isomer) from carrageenans (d-isomer) [18]. Apart from agarans [18], found in species of *Porphyra*, *Polysiphonia*, *Acanthophora*, *Goiopeltis*, *Bostrychia* or *Cryptopleura* (Table 2), red seaweeds are also good sources of κ-carrageenan (*E. spinosa*, *K. alvarezii*), λ-carrageenan (*Chondrus* sp, *G. skottsbergii* and *Phillophora*) (Table 2) [238], ι-carrageenan (*E. spinosa*) [239], and other heterogalactans with **man** and/or **xyl** bulding up their backbones. Among these, we may find xylogalactans in *N. fastigiata* [143], xylomannans in *S. polydactyla* [152] (Table 2).

Regarding green macroalgae, the information on their structures and applications is scarce. Nevertheless, Wangs’s group [8] has made an excellent overview on those properties for the **sPS** from several genera of “macro-chlorophytes”. These **sPS** are very diverse and complex, with various types of glycosidic bonds between monomers, and include galactans (*Caulerpa* spp.), rhamnans (*C. fulvescens* and *Enteromorpha*), arabino- and pyruvylated galactans (*Codium* spp.), and the most known ulvans from *Ulva* spp and *E. prolifera* (Table 3). Wang and coworkers [8] also included some repeating aldobiuronic di-units for the backbone of ulvans, containing **IduAc** or **glcAc** (*U. armoricana* and *U. rigida*, respectively), disaccharides (S-)**xyl-S-rham**, and a trisaccharide unit composed by 1,4-linked **glcAc**, **glcAc**, and S-**rham**. The backbone of rhamnans seems to be somewhat simpler (Table 3), but other types of glycosidic bonds can also appear. For example, four repeating disaccharide units were indicated for the homopolymer of *M. latissimum* [240] (Table 3). Species from *Codium* are very interesting: their **sPS** may include different percentages of arabinose (**ara**) and **gal**, giving place to arabinans (*C. adhaerens*; [153]), galactans (*C. yezoense*) [241], arabinogalactans [8]. Pyruvylated galactans were also identified in *C. yezoense* [241], *C. isthmocladium* [2] and *C. fragile* [242]. Some other species of *Codium* present other **PS**-types such as (1,4)-β-d mannans in *C. vermilara* [158], or the rare (1,3)-β-d mannans in *C. fragile* [243], with various sulphation patterns. *C. fulvescens* contains “vary branched” S-mannan as well [163].

### 2.2. Microalgae and Cyanobacteria

The characteristics of the various **PS** produced by microalgae, including their composition and structure, were recently discussed [14]. Some particular aspects about these polymers came to light. For example, it seems that concerning microalgae only *G. impudicum* and *C. vulgaris* contain homo-**PS** of galactose (**gal**) [23] and glucose (**glc**) [24], respectively, while the **PS** from the other species are heteropolymers of **gal**, xylose (**xyl**) and **glc** in different proportions. Rhamnose (**rham**), **fuc** and fructose can also appear, and some of the microalgal **PS** present uronic acids as well (Table 4). The glycosidic bonds are described for only a few **PS**, such as the one from *Aphanothece halophytica*, whose monosaccharides are mainly 1,3-linked, but linkages of type 1 also appear for **glc** and **glcAc** [230], which suggests that these two last molecules are terminal, and some multiple bonds, such as 1,2,4-linked and 1,3,6-linked mannose (**man**) residues [230], are present as well, suggesting some branches coming out from the backbone of the **PS**. Further, there are some special features of microalgal **PS**, as it is the case of acofriose 3-*O*-methyl-**rham** in the polymers of *Chlorella* [203], *Botryococcus braunii* and calcium-spirulan (**CaSp**) of *Arthrospira platensis* [244]. In *Porphyridium cruentum*, an aldobiuronic acid [3-*O*-(α-d-glucopyranosyluronic acid)-l-galactopyranose), or **glcAc-gal** disaccharide], and two hetero-oligosaccharides were also identified [245], and so did two other aldobiuronic acids [246], which were also found in other species of *Porphyridium* and *Rhodella* [247]. Furthermore, other repeating disaccharide-units [233,234], and some oligosaccharides were also highlighted [233]. In addition, Ford and Percival [196,197] found that the structure of the **sPS** from *Phaeodactylum tricornutum* was a ramified sulphated glucoronomannan, with a backbone composed by β-(1,3)-linked **man**; a triuronic acid, an aldobiuronic acid and a glucan made of β-(1,3)-linked **glc** were also identified as being constituents of the side chains of that polymer.

**Figure 1:**
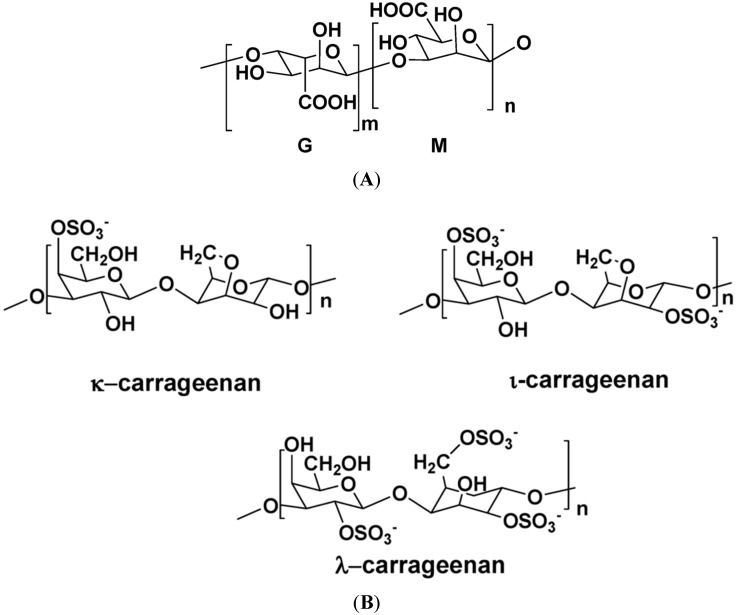

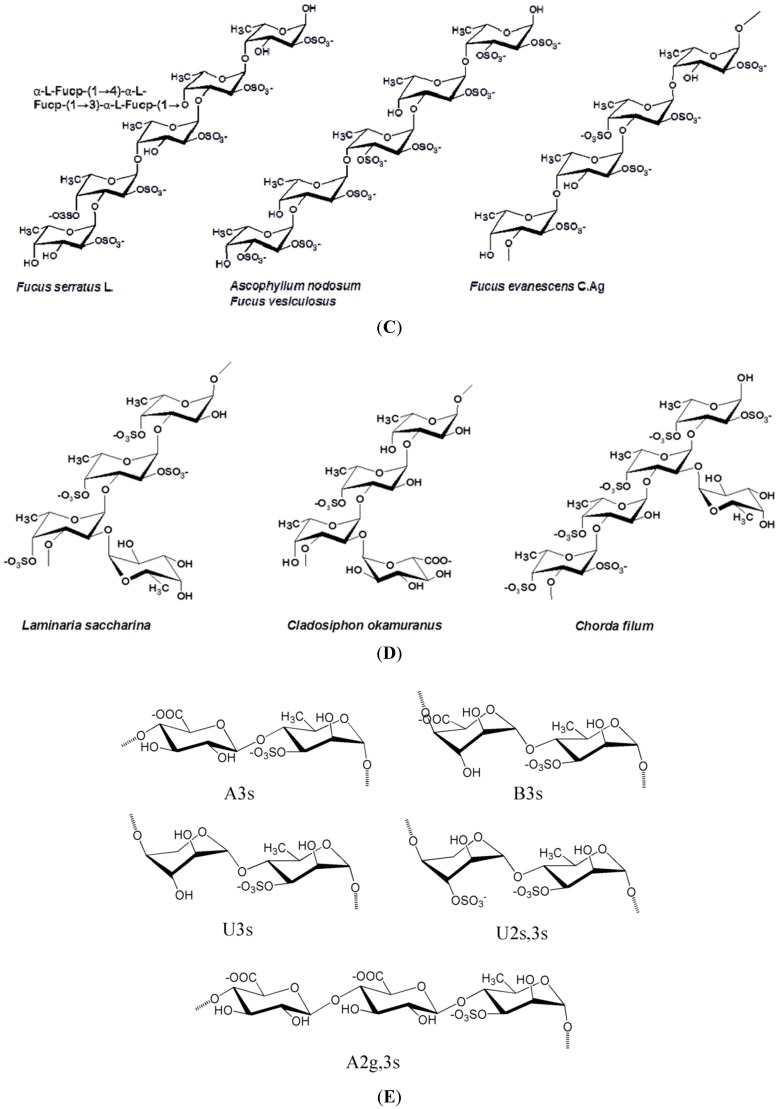

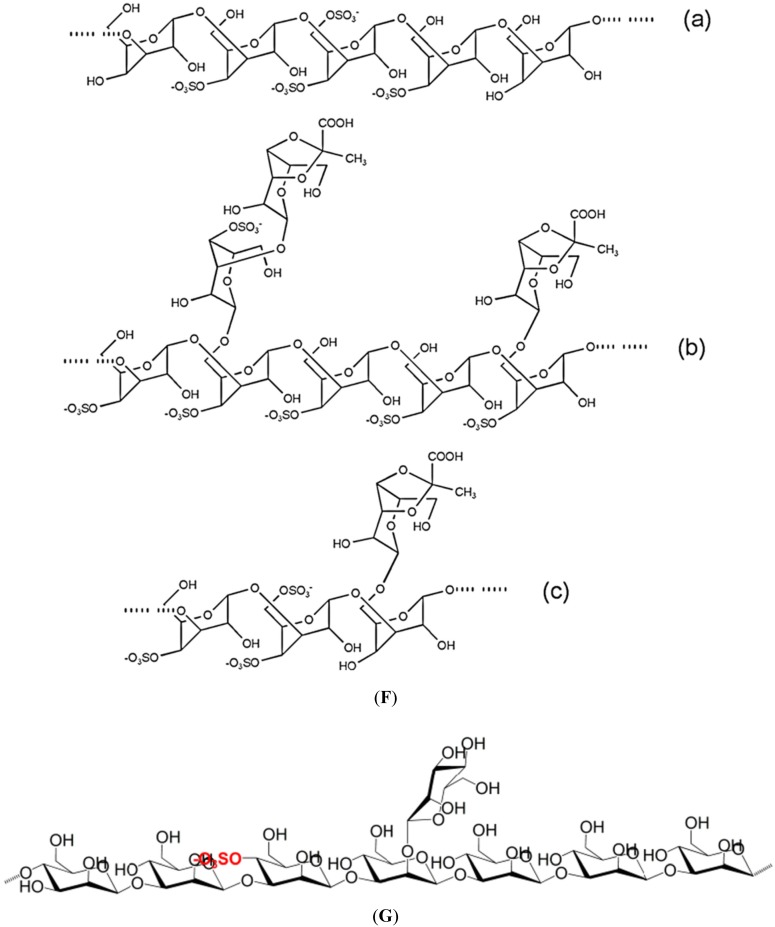

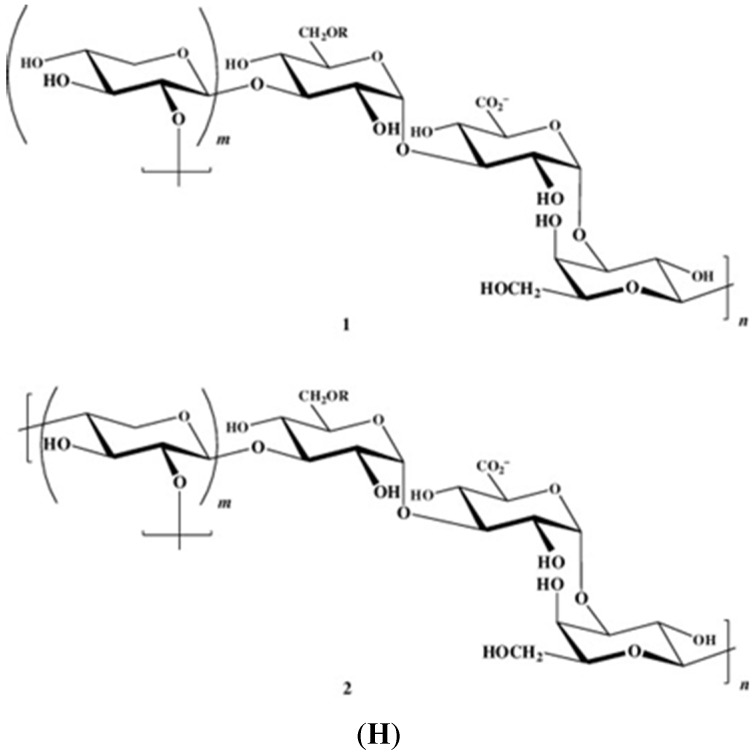
Examples of structures of **PS** from macro- and microalgae. (**A**) Repeating units suggested for the structure of alginates [3]; (**B**) Repeating units of some carrageenans [3]; (**C**) Fucoidan backbone of *A. nodosum* and three species of *Fucus*, showing the different distribution pattern of sulphate [75]; (**D**) Repeating units, sulphation pattern and gycosidic bounds of the backbone structures of PS of three different brown seaweeds [75]; (**E**) Alternative positions and combinations for the repeating units of ulvans. A3s and B3s are aldobiouronic repeating di-units suggested for *U. rigida* and *U. armoricana.* U3s and U2s,3s are, respectively, a xyl-(S-rham) and a (S-xyl)-(S-rham) disaccharides [8]; (**F**) Galactans of *Codium* spp. (a) linear (1,3)-β-d-galactan, (b) and (c) pyruvylated branched sulphated galactans [8]; (**G**) A rare mannan of the **PS** from *C. fragile*, with (1,3)-β-man residues and branches at C-2 [8]. Tabarsa *et al.* [243] referred that either branches or sulphates may be bound at the C-2 and/or C-4 positions along the PS backbone); (**H**) Models 1 or 2 for the possible acidic repeating unit in polysaccharide II, from *Porphyridium* sp. R = H, SO_2_O, terminal **gal** or terminal **xyl**, m = 2 or 3 [14].

Figure 1 illustrates the structures of some **PS** from macro- and microalgae.

## 3. Potential Medical/Biomedical Applications of Polysaccharides from Marine Algae. Relation with Some Chemical Features of Their Structures

The **PS** are complex and heterogeneous macromolecules, coming from different genera belonging to the larger groups of algae, and species and strains of the same genus. Often, difficulties are found in identifying their chemical structure and therefore, their biological activities not being thoroughly understood. Few researchers have focused on such a challenging task as the exploitation of possible relation chemical structure–activity of **PS**. One approach to look for structure–biological activity relationships has been to make inferences based on information obtained from studies of invertebrate sulphated polysaccharides that have a regular structure and, thus, could be more easily studied [18].

The types of glycosidic linkages and the contents and positions of the sulphate groups may be significantly different in the various **sPS**, depending on species, region of the thallus, growing conditions, extraction procedures, and analytical methods [2,186]. The biological and pharmacological activities of **sPS** normally result from a complex interaction of several structural features, including the sulphation level, distribution of sulphate groups along the polysaccharide backbone, molecular weight, sugar residue composition, and stereochemistry [248,249]. For instance, the general structural features of fucans that are important in their anticoagulation activity include the sugar composition, molecular weight, sulphation level and the position of sulphate groups on the sugar backbone [84,86,250,251]. Also, it has been observed that the antiviral activity of **sPS** increases with the molecular weight [252]. Galactans, fucans and galactofucans are representative polysaccharides from brown and red seaweeds that differ in structure, sulphation level and molecular weight, and yet all were shown to inhibit HSV-1 and HSV-2 infection [253]. Recently, by using NMR, it was found that branched fucoidan oligosaccharides might present higher imuno-inflammatory activity than linear structures, because they were better at inhibiting the complement system [254]. Usov [236] compared two sulphated galactans from *Botryocladia occidentalis* and *Gelidium crinale*. He concluded that the interaction of the **sPS** with different compounds participating in the coagulation process depends on the differences in the structural features; unfortunately, data on the configuration of galactose in the galactan from *G. crinale* are not sufficient to fully understand the relationship.

### 3.1. Antiviral, Antibacterial and Antifungal Activities

An overview on the antiviral activity against several kinds of virus and retrovirus, enveloped or naked was well documented by Carlucci *et al.* [255] and Wijesekara *et al.* [21]. These reviews focused on the HIV type 1 and type 2, the human papilloma virus (HPV), the encephalo-myocarditis virus, the hepatitis virus type A and type B and the dengue and yellow fever virus. The inhibition of infection by most of these viruses was explained by the action of **sPS**, which might block the attachment of virions to the host cell surfaces [140,256]. Another way of exerting their activity is by inhibiting the replication of the enveloped virus, such as the HIV, the human cytomegalovirus (HCMV) and the respiratory syncytial virus (RSV) [18,147,153], either by inhibiting the virus adsorption or the entry into the host cells. Some of the **sPS** are effective only if applied simultaneously with the virus or immediately after infection [18]. Another mechanism of action of fucoidans and other **sPS** is through the inhibition of the syncytium formation induced by viruses [21,257].

Some S-xylomannans were reported to present antiviral sulphate-dependent activity, as it was the case of **PS** from *S. polydactyla* and *S. latifolium*, which inhibited the multiplication of HSV-1 in Vero-cells [152,258]. In addition, the molecular weight (MW) seems to play an important role in the antiviral properties of the **sPS**, the effect increasing with the MW [18]. However, other structural features can be co-responsible for the reinforcement of the antiviral effectiveness, like sulphation patterns, composition and distribution of sugar residues along the backbone, and the complexity of the polymers [18,152,248,253]. Further, the fucoidans from *L. japonica* already proved their effectiveness in fighting both RNA and DNA viruses [103], such as poliovirus III, adenovirus III, ECHO6 virus, coxsackie B3 and A16 viruses. Moreover, these **sPS** can protect host cells by inhibiting the cytopathic activity of those viruses [99].

In addition to their virucidal activity against HIV and other viruses associated to sexually transmitted diseases (STD) [5], including HPV, some carrageenans might find application as vaginal lubricant gels and coatings of condoms, with microbicidal activity, for they do not present any significant anticoagulant properties or cytotoxicity [259,260]. Furthermore, some fucoidans, apart from inhibiting attachment of virus particles to host cells, were able to inhibit the attachment of human spermatozoids to the *zona pellucida* of oocytes [261]; this property could be used for the development of a contraceptive gel with microbicidal characteristics [20].

The polysaccharides produced by some marine microalgae, and which may be released into the culture medium, showed antiviral activity against different kinds of viruses, such as the HIV-1, HSV-1 and HSV-2, VACV and Flu-A (Table 4), as described by Raposo *et al.* [14]. Sulphated **PS**, in particular, proved to increase the antiviral capacity [231]. In fact, the antiviral activity of the **PS** may depend on the culture medium, algal strain and cell line used for testing, but also on the methodology, and the degree of sulphation, as is the case of **EPS** from *P. cruentum* [216,262]. Despite the slight toxicity that some **PS** may present, they could be safely applied in *in vivo* experiments, decreasing the replication of the virus VACV, for instance [223].

The mechanisms involved in the antiviral activity of **sPS** may be understood analyzing what happens when cells are infected by a virus. Just before infection, viruses have to interact with some glycosaminoglycan receptors (GAG), such as heparin sulphate (HS) [263]. The GAG to which a protein can be covalently bound are part of the target cell surface and can also be found in the intracellular matrix of various connective and muscle tissues. **SPS** may impair the attachment of the virus particles by competing for those GAG-receptors, as they are chemically similar to HS [130,255], most of them having a covalently linked core protein [264,265]. Besides, as it happens with GAG, **sPS** are negatively charged and highly sulphated polymers [40,255,266], whose monosaccharide distribution pattern might influence the specificity of the bound protein, determining several biological functions [263]. For viruses to attach to the host cell surface, the linkage between the basic groups of the glycoproteins of the virus and the anionic components of the **PS** (sulphate, for example) at the cell surface must be established [248]. In fact, whichever the algal **PS** is, either from seaweeds or microalgae, by mimicking these GAG, they may induce the formation of a virus-algal **PS** complex, thus, impairing the cell infection by blocking the interaction virus-host cell receptor. Hidari and coworkers [40], for instance, showed that dengue virus (DENV) establishes an exclusive complex with fucoidan, and viral infection is, therefore, inhibited. They suggested that arginine-323 had a high influence on the interaction between the DENV-2 virus and the fucoidan, in an *in vitro* experiment with BHK-21 cells. These researchers also found that glucuronic acid seems to be crucial since no antiviral activity was observed when this compound was reduced to glucose.

Sulphated polysaccharides from seaweeds, such as alginates, fucoidans and laminaran appear to have antibacterial activity against *E. coli* and species from *Staphylococcus*. A fucoidan from *L. japonica* and sodium alginate were found to inhibit *E. coli* [267], for example, by adhering to bacteria and killing those microorganisms [5], thus showing bactericidal properties. This type of **PS** is also a good antibacterial agent against *Helicobacter pylori*, eradicating their colonies, restoring the stomach mucosa, in clinical trial studies, and regenerating biocenosis in the intestines [268]. Laminaran from *Fucus*, *Laminaria*, *A. nodosum* and *U. pinnatifida* demonstrated to have an effect on pathogenic bacteria [56] as well, with the advantage of being unable to promote blood coagulation [269]. An S-galactan from *Chaetomorpha aerea* inhibited the growth of *Staphylococcus aureus* (50 mg/mL of extract) but not that of *Salmonella enteritidis* [270]. In contrast, the carrageenans from some seaweeds [271] and the sulphated exopolysaccharide (**sEPS)** from the red microalga *Porphyridium cruentum*, despite the higher concentration used [216], showed a significant inhibitory activity against *S. enteritidis*. In fact, some **PS** from microalgae, such as *A. platensis* (Table 4), may present antibacterial properties against some specific bacteria, the activity depending on the solvent used to extract the polymer, as referred to by Raposo *et al.* [14].

By stimulating the production and/or expression of ILs, dectin-1 and toll-like receptors-2 on macrophages and dendritic cells, respectively, (1,3)-β-glucans from, e.g., *C. vulgaris*, and laminarans, also induced antifungal and antibacterial responses in rats [272], and some resistance to mammal organisms towards infections by *E. coli* [273]. Therefore, these types of **PS** promise to be good antimicrobial agents.

### 3.2. Anti-Inflammatory and Immunomodulatory Activities

Polysaccharides from macro- and microalgae have long demonstrated to have biological and pharmaceutical properties, such as anti-inflammatory and immunomodulation (Table 1, Table 2, Table 3 and Table 4) [14]. Neverthless, the anti-inflammatory properties may be shown in several ways, depending on the **PS**, its source and type/site of inflammation. There is growing evidence that **sPS** are able to interefere with the migration of leukocytes to the sites of inflammation. For example, the heterofucan from *D. menstrualis* decreases inflammation by directly binding to the cell surface of leukocytes, especially polymorphonuclear cells (PMNs). It completely inhibits the migration of the leukocytes into the peritoneal cavity of mice where the injured tissue was after being submitted to simulated pain and inflammation, without the production of pro-inflammatory cytokines [1]. Sometimes, the recruitment of these PMNs shows to be dependent on P- and/or L-selectins, as it was demonstrated for fucoidans of some brown seaweeds [15,112].

Some other studies refer the association of the anti-inflammatory activity with the immunomodulatory ability. This seems to be the case in the work by Kang *et al*. [88], who simulated an inflammation process in RAW 264.7 cells (peritoneal macrophage primary cells) induced by lipopolysaccharides (LPS). They found that the fucoidan from *E. cava* inhibited, in a dose-dependent manner, the enzyme **n**itric **o**xide **s**ynthase **i**nduced by LPS (iNOS) and the gene expression for the enzyme cyclooxygenase-2 (COX-2) and, as a consequence, the production of nitric oxide (NO) and prostaglandin E2 (PGL2). Li *et al.* [274] confirmed the anti-inflammation mechanism *in vivo* via the immunomodulatory system *in vivo*, since the fucoidan from *L. japonica* reduced the inflammation of rats’ myocardium damaged cells, by inactivating the cytokines HMG B1 and NF-κB, two groups of proteins secreted by the immune cells during inflammatory diseases. These protective and regenerative effects of fucoidans (from *A. nodosum*), via the immunomodulatory system, were also verified in the destruction/proteolysis of connective tissue by Senni *et al.* [275]. These researchers referred to the fact that severe inflammation and the subsequent excessive release of cytokines and matrix proteinases could result in rheumatoid arthritis or chronic wounds and leg ulcers, which could be treated with fucoidans [275].

In addition to the polysaccharide from *Ulva rigida*, a green seaweed [192], the **sPS** p-KG03 from the marine dinoflagellate *G. impudicum*, also activates the production of nitric oxide and immunostimulates the production of cytokines in macrophages [227].

The enhancement of the immunomodulatory system by some **sPS** from marine algae is also a way for **sPS** to suppress tumour cell’s growth and their proliferation, and to be natural neoplastic-cell killers (apoptotic effect).

Studies with arabinogalactan and other fucoidans revealed them to be immunostimulators by activating macrophages and lymphocytes, which suggests their effectiveness in the immuno-prevention of cancer [22,276]. The **PS** from *U. pinnatifida* was also suggested to treat/relieve the symptoms of pulmonary allergic inflammation as it supresses the activity of Th2 immune responses [111]. On the other hand, fucoidan activated macrophages and splenocytes to produce cytokines and chemokines [277].

Polysaccharides from marine microalgae, such as *Porphyridium*, *Phaeodactylum*, and *C. stigmatophora* (Table 4), showed pharmacological properties, such as anti-inflammatory activity and as immunomodulatory agents, as reported by Raposo *et al.* [14]. Some of these **sPS**, for example, the ones from *C. stigmatophora* and *P. tricornutum* (Table 4), have revealed anti-inflammatory efficacy *in vivo* and *in vitro* [195]. The mechanisms underlying the anti-inflammatory and immunomodulatory activities may be unsderstood by making some considerations at the molecular level. On one side, the protein moiety that is covalently bound to most **PS** seems to play a critical role in the activation of NF-κB and MAPK pathways involved in the macrophage stimulation [265,278]. This was evidenced in an *in vitro* experiment performed by Tabarsa and colleagues [265]. They showed that the **PS** from *C. fragile* was not able to stimulate RAW264.7 cells to produce NO and the protein alone was also unable to induce NO release, but the complex **sPS**-protein did inhibit the inflammatory process. On the other side, several other researchers found that proteins were not essential or responsible for the immunostimulatory responses of the cells [192,279]. In addition, Tabarsa and coworkers [265] demonstrated that the sulphate content and the MW were not crucial for the stimulation of murine macrophage cells. In fact, both desulphated and LMW-PS derivatives of *C. fragile* produced immunomodulatory responses similar to the ones of the original **PS**. In contrast, the **sPS** from *U. rigida* induced a strong sulphate-dependent release of NO [192], thus, the sulphate content showing to be essential for the stimulation of macrophages. These researchers mentioned the possibility of the sulphate interfering in the interaction **PS**-cell surface receptors.

The interaction of algal **sPS** with the complement system suggests that they might influence the innate immunity to reduce the pro-inflammatory state [254]. In addition, algal polysaccharides have been shown to regulate the innate immune response directly by binding to pattern recognition receptors (PRRs) [280]. For example, λ-carrageenan stimulated mouse T cell cultures in a toll-like receptor-4 (TLR4) [281].

Different effects were observed in other types of **sPS**: Zhou *et al.* [282] proved that carrageenans from *Chondrus* with lower molecular weights better stimulated the immune system. The same trend was verified for the **sEPS** from the red microalga *Porphyridium* [221], a 6.53 kDa LMW-fragment at 100 µg/mL presenting the strongest immunostimulating activity.

It is worth remarking that carrageenans from red seaweeds are recognized for triggering potent inflammatory and carcinogenic effects either in rats and mice cells [130]. However, while some carrageenans stimulate the activity of macrophages, others inhibit macrophage activities [21].

Although **PS** from various macro- and microalgae do not show anticoagulant and/or antithrombotic activities, attention should be paid to the anticoagulant properties of some **PS**, since their use could cause severe bleeding complications. This issue will be discussed further on in this review.

### 3.3. Anti-Proliferative, Tumour Suppressor, Apoptotic and Cytotoxicity Activities

Because of the growing number of individuals suffering from different types of cancer and the secondary effects of synthetic chemicals and other types of treatment used against tumour damages, research was driven towards demand for natural therapeutics with bioactive compounds. In this context, **sPS** from both macro- and microalgae already proved to have antitumor biological activities.

An S-fucoidan from *C. okamuranus* exhibited anti-proliferative activity in U937 cells (myeloid cancer cell-line) by inducing cell apoptosis following a pathway dependent of Caspases-3 and -7 [43]. In another study, conducted by Heneji’s group [283], a similar fucoidan induced apoptosis in two different leukaemia cell lines. These results indicate that fucoidans might be good candidates for alternative therapeutics in treating adult T-cell leukaemia [22]. S-fucoidans from *E. cava* also seem to be promising to treat other types of human leukaemia (monocyte- and promyelocytic-origin) cell-lines [284]. There was some evidence that the fucoidan from *L. guryanovae* inactivated the epidermal growth factor (tyrosine kinase) receptor (EGFR), which is greatly involved in cell transformation, differentiation and proliferation [285,286]. Therefore, this kind of **sPS** could be used as antitumor and anti-metastatic therapeutical/preventing agent, which might act either on tumour cells or by stimulating the immune response [287].

Further, the **sPS** from *E. bicyclis* and several other seaweeds (Table 1, Table 2 and Table 3) have demonstrated their potent bioactivity against different kinds of tumours, including lung and skin, both *in vitro* and *in vivo* [62,83,288,289] causing apoptosis in various tumour cell-lines [62,290,291,292]. The mechanisms involved in this antitumor activity might be associated again with the production of pro-inflammatory interleukins IL-2 and IL-12 and cytokine interferon-gamma (INF-γ) by the immune-stimulated macrophages, together with the increase of the activity of the natural killer cells (NK cells) and the induction of apoptosis [62,293]. NK cells can also upregulate the secretion of IFN-γ, which can activate either the T-cells for the production of IL-2 or the macrophages, which, after being activated, keep on producing IL-12 and activating NK cells [293,294]. The enhancement of the cytotoxicity of these NK cells (lymphocytes and macrophages) can be stimulated by other **sPS** such as fucoidans and carrageenans from other seaweeds [276,282]. Polysaccharides can also activate some signalling receptors in the membranes of macrophages, such as Toll-like receptor-4 (TLR-4), cluster of differentiation 14 (CD14), competent receptor-3 (CR-3) and scavenging receptor (SR) [295]; these are also activated by other intracellular pathways, involving several other protein-kinases, that enhance the production of NO, which, in turn, plays an important role in causing tumour apoptosis [295]. These immunomodulation properties of S-fucoidans could be used for the protection of the damaged gastric mucosa as it was already demonstrated by using rat-models [296]. More information on the pathways and mechanisms responsible for the immune-inflammatory activities, including the involvement of the complementary system, may be found in Jiao and colleagues’ work [18].

The anti-adhesive properties of some **sPS**, especially fucoidans might also explain their anti-metastatic activity (Table 1, Table 2 and Table 3), both *in vitro* and *in vivo*, in various animal-models [15,297], as they can inhibit the adhesion of tumour cells to platelets, thus decreasing the possibilities of proliferation of neoplastic cells. The mechanisms by which fucoidans and other **sPS** exert their anti-adhesive ability were well documented by Li’s group [103]. Some researchers also highlighted the mitogenic properties and the cytotoxicity and tumoricidal activity of some arabinogalactans and fucoidans as well [42,276], either in different cell-lines or various animal-models.

The anti-adhesive properties of algal **sPS** may also be relevant as these polymers can block the adhesion of tumour cells to the basal membrane, thus demonstrating to impair implantation of tumour cells and metastatic activity by binding to the extracellular matrix [37]. For example, the **sPS** from *Cladosiphon* was shown to prevent gastric cancer *in vivo*, since it inhibited the adhesion of *H. pylori* to the stomach mucosa (mucin) of gerbils [45]. Metastasis appearance could also be reduced *in vivo* by S-laminaran, a 1,3:1,6-β-d-glucan, because this compound inhibited the activity of heparanase, an endo-β-d-glucuronidase involved in the degradation of the main **PS** component in the basal membrane and the extracellular matrix. The expression of this enzyme is known to be associated with tumour metastasis [59].

These antitumor properties may also be found in some **PS** from microalgae, such as *A. platensis*, which are inhibitors of cell proliferation [234]. Other **sPS**, such as **sPS** p-KG03 from *G. impudicum*, have also anti-proliferative activity in cancer cell lines (*in vitro*) and inhibitory activity against tumour growth (*in vivo*) [227,228,298]. Other **PS** from microalgae, such as *C. vulgaris* (Table 4), and **sPS** or LMW-derivatives of **sPS** from *P. cruentum* (Table 4), for example, are described as having similar properties in the review performed by Raposo *et al.* [14].

In some research work, the immunomodulatory activity was associated to the ability of inhibiting carcinogenesis. For example, Jiao’s group [172] found that a sulphated rhamnan and some derivatives from the green seaweed *E. intestinalis* suppressed tumour cell growth *in vivo* (mice), but they did not show any toxicity against tumour cells *in vitro*. The oral administration of the **sPS** to mice enhanced the spleen and thymus indexes, and also induced the production of TNF-α and NO in macrophages, increased lymphocyte proliferation, and enhanced TNF-α release into serum.

The degree of sulphation may play some role in the carcinogenesis process, although the action of the **sPS** may also depend on the type of tumour. In fact, an oversulphated **PS** demonstrated the capacity of inhibiting the growth of L-1210 leukaemia tumour in mice, but, on the other hand, it was unable to inhibit the growth of Sarcoma-180 tumour in mice [83]. In addition to the sulphation level, MW may also influence the anticancer activity. For instance, LMW-PS derivatives showed to enhance antitumor activity [91,299]. However, the increment in the anticancer activity greatly depends on the conditions of the **PS** depolymerisation [299]. Kaeffer *et al.* [186] suggested that the *in vitro* antitumor activity of LMW-PS, sulphated or not, against cancerous colonic epithelial cells (Caco cells) might be associated with the inhibition of tumour cells proliferation and/or differentiation.

### 3.4. Anticoagulant and Antithrombotic Activities

There are several studies on the anticoagulant properties of **PS** isolated from seaweeds, presented in a recent review [14] by different groups of researchers: Wang *et al.* [8], Costa and colleagues [2], Cumashi *et al.* [15], Athukorala *et al.* [300] and Wijesekara and coworkers [21].

The main sources of the **sPS** from green seaweeds with anticoagulant properties are *Codium* and *Monostroma* [167,301]. Some of the **PS**, such as S-rhamnans, showed their action by extending the clotting time via the intrinsic and extrinsic pathways [167]. In fact, *Codium* spp present strong anticoagulant effects [159,160], but other species from Division/Phyllum Chlorophyta also contain **sPS** (native, low-molecular or otherwise modified) with anticoagulant properties (Table 3). The mechanism of action of the referred **PS** is mostly attributed to either a direct inhibition of thrombin or by enhancing the power of antithrombin III [302,303].

Some other **PS** from green seaweeds also showed potent anticoagulant properties but their mechanisms of action are associated not only to a direct increase in the clotting time (APTT assays) by inhibiting the contact activation pathway (intrinsic pathway), but also by inhibiting the heparin cofactor II-mediated action of thrombin [180,304] thus showing a potent antithrombotic bioactivity.

In addition to their anticoagulant properties demonstrated *in vitro* by APTT and TT tests, several **sPS** from algae of different groups (Table 1, Table 2 and Table 3) present antithrombotic qualities *in vivo* [305,306] by increasing the time of clot formation. In fact, Wang and colleagues [8] published an exhaustive work on this issue by including a summary table with 24 references about both the anticoagulant, and anti- and prothrombotic activities of several **sPS** from various green seaweeds. In two other studies, Wijesekara *et al.* [21] and Costa and coworkers [2] also included the **sPS** from brown and red macroalgae that present effects on the blood clotting time. Wijesekara and colleagues [21] referred to the fact that there are few reports on the interference of **PS** from algae on the PT (prothrombin) pathway, meaning that most of the marine **sPS** may not affect the extrinsic pathway of coagulation [21]. As a matter of fact, Costa *et al.* [2] did not detect any inhibition in the extrinsic coagulation pathway (PT test), for the concentrations used; only *C. cupressoides* increased the clotting time. In addition, they found no anticoagulant properties (APTT and PT assays) in the **sPS** from a brown seaweed (*S. filipendula*) and a red macroalga (*G. caudate*). Further, in our laboratory we found no anticoagulant properties in the **sEPS** from different strains of the red microalga *P. cruentum*, despite the high content in sulphate and molecular weight. As Costa *et al.* [2] observed, this could be due to the absence of sulphate groups in the monosaccharides at the non-reducing ends of the branches, which impaired the interaction between target proteases and coagulation factors. Nishino *et al.* [84] and Dobashi *et al.* [72] defended that there might be no effect above an upper limit for the content in sulphate, since the difference in the anticoagulant and antithrombotic activities decreased with the increase of the sulphate content.

It seems that some of the chemical and structural features of the **sPS** may have some influence on their anticoagulant and/or antithrombotic activities. The degree and distribution pattern of sulphate, the nature and distribution of monosaccharides and their glycosidic bonds, and also the molecular weight showed to play some role on the coagulation and platelet aggregation processes induced by S*-*galactans and S-fucoidans [2,307,308]. In fact, at least for some fucoidans, the anticoagulant properties are related to the content in C-2 and C-2,3 (di)sulphate, this last feature being usually common in these **PS** [52,53,105]. Several other studies documented the anticoagulant activity and inhibition of platelet aggregation [22,103,130], supplying more information on the mechanisms of different **sPS** for these biological activities. Higher MW-**PS** usually present stronger anticoagulant activity [309] and if a **PS** has a more linear backbone, a longer polymer is required to accomplish the same anticoagulant effects [251]. However, both the native **PS** and LMW-derivatives of the green seaweed *M. latissimum* presented strong anticoagulant activities [168]. Nishino and colleagues also observed that high molecular weight fucans (e.g., 27 and 58 kDa) showed greater anticoagulant activity than the ones with lower molecular weight (~10 kDa) [85]. They found that a higher content of fucose and sulphate groups coincided with higher anticoagulant activities of sulphated polysaccharide fractions from *E. kurome* [84]. However, despite its high sulphation level, the galactofucan from *U. pinnatifida* lacks significant anticoagulation activity [38]. Moreover, an S-galactofucan from the brown seaweed *S. schröederi* did not present any anticoagulant properties *in vitro*, but demonstrated a strong antithrombotic activity when administered to an animal-model during an experimental induced venous thrombosis, this effect disappearing with the desulphation of the polymer [38].

As for other **PS**, the anticoagulant properties of the **PS** from marine microalgae may not only depend on the percentage of sulphate residues, but rather on the distribution/position of sulphate groups and, probably, on the configuration of the polymer chains [14]. Spirulan from *A. platensis* (Table 4) is one of the **PS** from marine microalgae that strongly interferes with the blood coagulation-fibrinolytic system and exhibits antithrombogenic properties [159,310], therefore, promising to be an anti-thrombotic agent in clots’ breakdown, although care should be taken regarding hemorrhagic strokes [14].

It seems that the anticoagulant mechanisms of action of **PS** may be attributed to: (i) the inhibition of thrombin directly or via antithrombin III (AT-III) [66,302,303,311,312]; (ii) the increment in the activity of thrombin inhibitors, such as AT-III and/or heparin cofactor II (HC-II) [130,304,313], in both the intrinsic (contact activation or normal, measured by APPT test) and extrinsic (Tissue factor, TF, measured by PT test) pathways [314], the activation of HC-II seeming to be sulphate-dependent [315].

One explanation for the **sPS** to act directly on thrombin may be associated with the ability of those polymers to bind to thrombin, thus, hindering its catalytic activity [15,316]. In addition, some **sPS** may also inhibit thrombin from linking to their receptors in human platelets (protease activated receptor-1 and GP-1b) [317]. However, a high content of glucuronic acid might render a **sPS** unable to interfere in the coagulation process [15].

### 3.5. Antilipidaemic (Hypocholesterolaemic and Hypotriglyreridaemic), Hypoglycaemic and Hypotensive Activities

Sulphated **PS** from seaweeds are potent inhibitors of human pancreatic cholesterol esterase, an enzyme that promotes its absorption at the intestinal level; this inhibitory effect is enhanced by higher molecular weights and degree of sulphation [6].

An S-ulvan from *U. pertusa* in an *in vivo* study using mice-models regulated the ratio HDL/LDL-cholesterol and reduced the levels of triglycerides (TG) in serum [185]. However, in another experiment with rats and mice, using native ulvans from the same species, the animals experienced a hypocholesterolaemic effect but no reduction in the TG profile [318]. An opposite reaction was observed when the **PS** was acetylated and oversulphated, as TG levels were normalized. It seems that the ability to sequester bile extracts may be involved [185]. The contents in sulphate and acetylate groups play important roles during the dislipidaemia process [191,319]. Ulvans from *Ulva* spp also showed antiperoxidative properties, preventing liver tissues from hyperlipidaemia, including that induced by toxic chemicals and protecting the injured tissue from the oxidative stress [189], and improving antioxidant performance of the animal models. In fact, these **sPS** regulated superoxide dismutase (SOD) and catalase, increased vitamins E and C, and reduced-glutathione, and had some role in reducing the levels of aspartate and alanine transaminases in the rats’ liver [179,185]. Further, the **sPS** from *M. nitidum* also demonstrated hepatoprotective activity by increasing the expression of liver detoxifying enzymes, and, therefore, showed to be good agents for chemoprevention medicine [171]. The activity of these **PS** may be related to their uronic acid and sulphate content, which are able to sequester and bind to bile acids [320], reducing their levels. Other **sPS** from green seaweeds also revealed hypolipidaemic properties, such as that from *E. prolifera*. This **PS** regulated the lipidic profile both in plasma and liver, increasing HDL-cholesterol, in rats [177]. Fucoidans from *L. japonica*, the native or LMW-derivate, have hypolipidaemic effects, decreasing total and LDL-cholesterol in the serum and TG in rats [321], and they prevented hypercholesterolaemia in mice [322]. Another mechanism to reduce blood cholesterol in humans by **sPS** is associated to their high capacity to inhibit pancreatic cholesterol esterase, which is responsible for the absorption of cholesterol and fatty acids at the intestine [6]. It seems that the presence of sulphate at the C-3 position of the sugar residues greatly enhances that inhibition [6]. Porphyran from *P. yezoensis* has anti-hyperlipidaemic properties [119,323] by reducing the release of apolipoprotein-B100 (apoB100) and decreasing the synthesis of lipids in human liver cultured cells [324]. By reducing the secretion of apoB100, porphyran has the potential to be used as a therapeutic agent to treat CVD. In addition, some types of carrageenans have already proved to decrease blood cholesterol in humans [325] and in rats fed on a diet enriched with a mixture of κ/λ-carrageenans from *G. radula* [326].

Most of the **PS** from marine microalgae are naturally highly sulphated, with high molecular weights, making them not-easily absorbable and thus enabling them to be used as anticholesterolaemic agents. Few studies were carried out in this area, namely focusing on *Porphyridium*, *P. cruentum*, *R. reticulata* (Table 4) [327,328,329,330], but these suggest a strong potential of sulphated polysaccharides from unicellular algae to be used as hypolipidaemic and hypoglycaemic agents, and as promising agents for reducing coronary heart disease, due to their hypocholesterolaemic effects [14].

As far as we know, scarce research was performed on the mechanisms underlying the antihyperlipidaemic activity. However, the sequestration and disruption of the enterophatic circulation of the bile acids may be involved [185,331,332]. For example, ulvans and their LMW-derivatives, and also the **sEPS** from *Porphyridium* showed to increase the excretion of bile [185,333]. Another explanation for the antihyperlipidaemic activity of **sPS** may be associated to the fact that they can effectively increase the anionic charges on the cell surface, which improve the removal of cholesterol excess from the blood, thus, resulting in a decrease of serum cholesterol [103]. In addition, most **PS** have ion exchange capacity, such as those from *Porphyridium* and *Rhodella* [334], and they can function as dietary fibres. This could also explain the ability to lower down cholesterol [335]. **PS** may act as dietary fibres, immunostimulating the goblet cells in the intestine to increase the release and effects of mucin [336]. Moreover, the administration of **PS** may increase the viscosity of the intestinal contents, interfering with the formation of micelles and nutrient absorption, thus, lowering lipid absorption, and reducing gastrointestinal transit time (GTT) [333,337].

Other **PS** have the ability to inhibit the enzyme α-glucosidase, thus improving the postprandial hyperglycaemia [338], and another can also reduce the blood pressure by inhibiting the release of plasma angiotensin II [339].

### 3.6. Antiaging (Antioxidant) Activity

The main mechanism by which **sPS** from green seaweeds exert their primary antioxidant action is by scavenging free-radicals (superoxide, hydroxyl, 1,1-diphenyl-2-picrylhydrazyl (DPPH)-radicals) or by inhibiting their appearance [8]. They also demonstrated to have total antioxidant capacity, and a strong ability as reducing agents and as ferrous chelators [8]. However, some other **sPS**, such as S-heterogalactan (*C. cupressoides*) do not show a good scavenging power, but they are rather powerful against reactive oxygen species (ROS) [340]. It is interesting to note that fucoidans from brown seaweeds seem to exert a reducing power bigger than the **sPS** from other groups [2]; the **PS** from *S. filipendula* has an effect even stronger than vitamin C. Moreover, the fucoidan from *L. japonica* has a great potential to be used in medicine in order to prevent free-radical-mediated diseases, as it successfully prevented peroxidation of lipids in plasma, liver and spleen *in vivo* (mice), despite showing no effects *in vitro* [100]. The **sPS** from another species of *Sargassum* (*S. fulvellum*) has shown a NO scavenging activity higher than some commercial antioxidants [341]. In addition, the **sPS** from the red macroalga *P. haitanensis* demonstrated to decrease antioxidant damages in aging mice [115].

It seems that LMW-**sPS** may present higher antioxidant activity than the native polymers, as it was verified with the **PS** from *U. pertusa* and *E. prolifera* [166,342]. It is probably related with the ability of **PS** to be incorporated in the cells and to donate protons [21].

As noted by Raposo *et al.* [14], sulphated **PS** produced and secreted out by marine microalgae have shown the capacity to prevent the accumulation and the activity of free radicals and reactive chemical species. Therefore, **sPS** might act as protecting systems against these oxidative and radical stress agents. The **sPS** from *Porphyridium* and *Rhodella reticulata* (Table 4) exhibited antioxidant activity [343,344], although some research revealed no scavenging activity and no ability to inhibit the oxidative damage in cells and tissues for the crude **sPS** with high molecular weight from *Porphyridium cruentum*, while the **EPS**-derived products after microwave treatment showed antioxidant activity [220]. In all cases, the antioxidant activity was dose-dependent. Methanolic extracts of **EPS** from *A. platensis* also exhibit a very high antioxidant capacity [235].

Due to their strong antioxidant properties, most of the **sPS** from marine macro- and microalgae are promising since they may protect human health from injuries induced by ROS, which can result in cancer, diabetes, some inflammatory and neurodegenerative diseases, and some other aging-related disorders, such as Alzheimer and CVD.

The influence of sulphate content on the antioxidant activity depends rather on the origin of the **PS**. For example, the **PS** from *U. fasciata* and other macro- and microalgae with lower sulphate content demonstrated a strong antioxidative power [165,181,220,343], while the antioxidant activity observed in **PS** from *E. linza* and other seaweeds showed to be sulphate-dependent [174,345]. Furthermore, high sulphated **PS** was shown to have an enhanced scavenging power [97,182], this property being also dependent on the sulphate distribution pattern [2]. It seems, in addition, that the protein moiety of **PS** may play some role on the antioxidative power. For example, Tannin-Spitz *et al.* [343] reported a stronger antioxidant activity for the crude **PS** of *Porphyridium* than for the denatured **PS**.

Zhao *et al.* [346] found that the antioxidant activity of **sPS** was apparently related, not only to molecular weight and sulphated ester content, but also to glucuronic acid and fructose content. This antioxidant activity seems to be attributable to metal chelating, free radical and hydroxyl radical scavenging activities of the **sPS**.

### 3.7. Nutritional Applications: Fibres (Dietary), Prebiotic and Probiotic

As already mentioned by Raposo *et al.* [14], **PS** can find applications in the food industry as emulsifying and gelling agents, as flocculant and hydrating agents, emulsifiers, stabilizers, thickening agents, *i.e.,* food additives [347], like agar E406, alginates E400-404, carrageenan E407. The s**PS** from marine microalgae could be used as nutraceuticals due to their content in fibres, the ability of acid binding and for cation exchange, and the properties of faecal bulking as well, being also good candidates as prebiotics [348]. The **PS** alone or in combination with other compounds have a great potential to be used in edible films and coatings of foods, while carriers of flavors, colorants, spices and nutraceuticals [349]. In our laboratory, experiments have already been carried out with based **EPS** from *P. cruentum*-coatings applied to fresh-cut apple. These polymers also have the potential to be used in low-fat or fat-free food products, as fat substitutes in mayonnaises [350,351], salad dressings and other food emulsions [352].

### 3.8. Other Biological Activities

As it happens in relation to the fucoidan from *S. schröederi* (Dictyotales) [36], a heterofucan-derivative from *D. menstrualis*, another member of Dictyotales, also presented antinociceptive activity. It acted as a peripheral analgesic agent, reaching 61.2% of pain reduction (4 mg/kg) in mice, this effect being as potent as dipyrone’s, and it was dose-dependent [1]. This suggests that this kind of S-fucans and some S-galactans can act as analgesic agents but not as anaesthetic ones, as they do not decrease pain when it involves the CNS. S-galactan from *G. cornea* is another **sPS** with analgesic characteristics, but at a higher concentration (9 mg/kg) [353]. A S-galactan from *C. feldmannii* is a more potent antinociceptive agent (80% reduction in contractions), but it also presents good anticoagulant properties [354]. Sulphated **PS** from *C. cupressoides* [155,156], at a dose of 27 mg/kg/day, reduced by 90% the writhes induced in mice by acetic acid, but they also showed analgesic effects only via peripheral mechanisms [156]. It seems that these **sPS** act by binding to the surface of the leukocytes, hindering their migration to the focus of tissue injury [1,355], therefore, demonstrating anti-inflammatory properties as well. Thus, all these **sPS** promise to be good peripheral antinociceptive agents, with some special care in relation to the galactan from *Champia feldmannii* due to its anticoagulant properties.

The angiogenic (neovascularization) properties of **PS** can be considered according to two angles. When dealing with treatment/prevention of neoplasias it is very important that the **PS** in question does not show that ability, so that the tumour will be reduced, and cells might die if not irrigated. Therefore, **sPS**, such as fucoidans may function as tumour supressors by inhibiting angiogenesis induced by tumour cells [3]. However, if the disorder we are dealing with is the result of an ischaemic issue, a **PS** with angiogenic activity should be used in order to re-establish the blood flow of the injured tissues, thus, acting as cardioprotective after ischaemia. The angiogenic mechanisms of fucoidans and glucans were well explained by Fedorov *et al.* [3] and Cumashi *et al.* [15].

Angiogenesis involves the differentiation of mature endothelial cells, their proliferation and migration. In fact, some **sPS** demonstrated the capacity to promote therapeutic revascularization in animal models, increasing the vessel formation when administered by injection in rats with ischaemic hind limb [57]. The mechanisms involved in the angiogenic properties of modified fucoidans are associated with the ability of these polymers to interact with endothelial cells, modulating the activity of proangiogenic growth factors, such as fibroblast growth factor-2 (FGF-2). The latter is mitogenic for that type of cells, fibroblasts and smooth muscle cells [103], and extracellular matrix components [58,356,357]. In fact, there is a correlation of the reduction of plasminogen-activator inhibitor (PAI-1) secretion with the upregulation of cell-surface α-6 integrin sub-unit. This could be an explanation for the proangiogenic ability, including the induction *in vitro* of tube formation by human endothelial cells. The fucoidans of *C. okamuranus* and *F. vesiculosus* are promising in treatment of ischaemic disorders, including infarcted myocardium, as they did not show to inhibit tubulogenesis in HUVEC cells. This cardioprotective activity was confirmed in animal models by enhancing creatinine phosphokinase, lactate dehydrogenase, and alanine and aspartate transaminases [47].

Fucoidans from two species of *Laminaria* and three species of *Fucus* revealed antiangiogenic properties, through the inhibition of the *in vitro* neogenesis of tubules in human umbilical vein endothelial cells (HUVEC), while a decrease in PAI-1 in HUVEC supernatants was also observed [15]. It is worth noting that these **sPS** revealed anticoagulant and antithrombotic activities, and some of these fucoidans inhibited the adhesion of breast cancer cells to platelets, as well, thus showing anti-adhesive and anti-metastatic properties. These features suggest that this type of polymers could be used as complementary agents in the therapeutical treatment of cancer.

In addition to the cardioprotective effects, the fucoidan from *C. okamuranus* Tokida demonstrated a great potential to be used as a gastroprotective agent [46]. It was used as a component of a new drug to treat/prevent gastric ulcers, and to inhibit *Helicobacter pylori* from adhering to the mucosa of the stomach [358], and also inhibited stomach cancer [44].

The fucoidans from other seaweeds are promising as well, not only as hepatoprotective agents against chemical damages, stimulating the release of IL-10 and inhibiting proinflammatory cytokines [359,360], but also against hepatic fibrosis, protecting hepatocytes and inhibiting the proliferation of hepatic stellate cells, which are co-responsible in the process [361].

Being an antioxidant against free radicals, fucoidan from *F. vesiculosus* might be an alternative or complementary therapeutic in uropathy and renalpathy, since it could prevent from the injuries caused by oxalate-induced free radicals [362] and from the mitochondrial damages associated to the process [363]. Several other disorders of the urinary system, including Heymann nephritis, are also liable to treatment or complementary therapeutics through the use of fucoidans [293,364,365,366,367].

The **PS** from other seaweeds demonstrated either stimulatory or inhibitory effects on some enzymes as was reported in the review by Smit [20], and inhibited cytotoxic and myotoxic effects against snake venoms as well, thus protecting the muscle from necrosis [368].

### 3.9. Biomedical Applications

Biomedical field is constantly demanding for new biomaterials with innovative properties. Natural polymers appear as materials of election for this objective due to their biocompatibility and biodegradability [369].

Alongside their biological activity and potential pharmaceutical use, as has already been addressed in this review, **PS** may be used as biomaterials, as such, or in combination with other synthetic or natural substances. There are several potential biomedical applications for **PS** in: regenerative medicine, such as wound management products, drug delivery systems (DDSs), tissue engineering, and medical fibres and biotextiles [369,370] (Table 5).

**Table 5:** Some applications of algal **PS** in biomedicine.

Groups of PSs	Possible Sources	Applications	References
Alginates	*Laminaria* spp, *A. nodosum*, *Ecklonia* sp., *M. pyrifera*, *Durvillaea*, *Lessonia*	Drugs carriers	[371]
		Encapsulation	[372,373,374]
		Scaffolds for ligaments and tissue engineering	
		Regeneration of tissues	
		Moulding in dentistry	
		Wound healing and dressings	[375,376,377]
Agaroids	*B. montaignei*, *Goiopeltis* spp., *A. tenera*, *P. capillacea*	Cell encapsulation	
		Scaffolds for tissue engineering	[378]
		Wound healing and dressings	[379]
		Revascularization	[380]
Ulvans	*Ulva rigida*, *Ulva* spp.	Drug carriers	[381]
		Wound dressings	[382,383]
		Tissue engineering	[384]
β-glucans	*A. nodosum*, *E. bicyclis*, *Fucus* sp., *Laminaria* sp., *U. pinnatifida* (laminaran); *C. vulgaris*	Wound healing	[385,386,387]
		Burn-wound dressings	
		Tissue regeneration	[388,389,390]
fucoidans	*U. pinnatifida*	Vaccines for immunotherapy	[299]
**PSs** from microalgae	*A. platensis*	Production of nanofibers	[391]
		Gluing and soft tissue closure after surgery	[6]
	*Porphyridium*	Lubricants for bone joints	[212,392]

Alginates from macroalgae have been most used in several applications: controlled drug release [371], cell encapsulation, scafold in ligament, tissue engineering and regeneration of almost all tissues in the human organism, or even preparation of moulds in dentistry [372,373,374]. A review recently made available was devoted to the processing of alginate fibres for use as wound management materials [375,376]. **PS** have been widely applied as hydrogels, eventually combined with other substances, for: the encapsulation and delivery of Langerhans islets [393], ovarian follicles [394] and stem cells in neural tissue engineering [395], skin tissue engineering [396], bone tissue engineering [397] and skeletal muscle regeneration [398]; regenerating the osteochondral interface [399]; capturing of endothelial progenitor cells from the human blood [400]. Kaltostat^®^ is a commercially available alginate dressing that promotes haemostasis and manages exudate in low to moderately exuding wounds. In the form of porous scaffolds, alginate has been used for creating a capillary bed in newly reconstructed tissues [401], and in the form of electrospin nanofibrous scaffolds, for constructing vascular replacements containing endothelial cells and smooth muscle cells (SMC) [402]. Alginate may also be used as a component of scaffolds for heart valve engineering [403], and for cardiac tissue engineering [404].

Similarly to alginate, agar can be used for cell encapsulation in tissue engineering applications. Due to its chondrogenic potential, agar was selected to entrap chondrocytes within poly-l-lactide scaffolds [378]. Composite membranes with agar proved to be promising wound dressings for healing burns or ulcers [379]. Agar gel supported the formation of *in vivo* autologous vascular prosthetic tissues, called “biotubes” [380].

However, not all polysaccharides are suitable for tissue engineering, mainly due to their jelly-like consistency and insufficient mechanical properties. As referred above, even **PS** used in tissue engineering usually need to be combined with other natural or synthetic polymers, or reinforced with inorganic substances [405].

A new generation of medical textiles incorporated with **PS**, such as alginate, is growing with respect to, in wound management products [406]. The main qualities of fibres and wound dressing products include antiviral, fungistatic, non-toxic, highly absorbent, non-allergic, breathable, haemostatic, and biocompatible. Such products with good mechanical properties may incorporate medication [377].

**sPS** are capable of binding to protein and may be involved in the cell development, cell differentiation, cell adhesion, cell signaling and cell matrix interactions. These bioactive molecules present a great potential for medical, pharmaceutical and biotechnological applications, such as wound dressings, biomaterials, tissue regeneration and 3D culture scaffolds, and even drugs [19]. Their biological activities and their resemblance to glycosaminoglycans (GAGs), which have been most studied, might position these **PS** in advantage. For example, ulvans from green algae, may be processed into porous structures, including nanofibres [381], particles [382], membranes [383] and hydrogels [384], which make them good candidates for medical applications, such as drug delivery, wound dressing and bone tissue engineering [381,382,383,384]. Carrageenans, besides being hydrogels, may also be processed into fibres, membranes or porous structures for several biomedical applications [407].

Fucoidans from brown algae besides having application in the biopharmaceutical industry (immunomodulatory, antiviral and anticoagulant agents), have found new applications in biomedicine, for instance, as nanoparticles of fucoidan and chitosan [408], and more recently in the synthesis of biohybrid glycopolymers [409].

The (1,3)-β-glucans have been used to help healing wounds, by inducing the migration of macrophages to the wound site [385], to accelerate the healing process as a constituent in composites [386,410], and in burn-wound dressings, therefore, reducing the need for analgesics [387]. Fucoidan-chitosan films and/or gels may be used to treat dermal burns and regenerate epithelial tissue [388]. The mechanism of action of hydrocolloids may be associated to the fact that wound dressings with **sPS** decrease wound secretions and scars, reducing the bacterial inflammation [389,390], with the advantage of being free from prions or other animal contaminants.

Native **sPS**, LMW- or otherwise modified derivatives, are also promising in the prevention of arteriosclerosis after cardiac transplantation [411,412], in coating encapsulates for controlled drug delivering, as new materials for cell immobilization and tissue engineering [7,413], and as carrier-materials for transplantation of chondrocytes and osteoblasts, improving neo-cartilage and neo-bone formation [414].

The use of fucoidans in dendritic cell-based vaccines for cancer immunotherapy has been also suggested [299].

Another promising and emerging application of the **PS** from microalgae might be associated to the production of nanofibres from the biomass of *A. platensis* (Table 4), to be used as extracellular matrices for the culture of stem cells in order to treat spinal cord injuries [391].

Their gluing and adhesive capacities, as well as their strong cohesive and binding strength, allied to their non-toxic and non-irritating properties, make the bioadhesive **PS** produced by marine microalgae good candidates as mucobioadhesives or glues for bone gluing and soft tissue closure after surgery. They might also replace the metallic screws and traditional wound closure methods, respectively [6].

The **sEPS** from *Porphyridium* (Table 4) has already shown a good lubrication capacity [355], being an excellent candidate to substitute for hyaluronic acid as a biolubricant. Another promising application could be as a component of a joint-lubricating solution, as it was demonstrated by injecting the **EPS** from *Porphyridium* into the joints of rabbits’ knees [212], thus mitigating degenerative joint disorders caused by arthritis.

## 4. Conclusions

Polysaccharides may be regarded as key ingredients for the production of bio-based materials in life sciences (e.g., medical devices, pharmaceutics, food, cosmetics). There are an enormous variety of polysaccharides that can be synthetized and/or released by marine macro- and microalgae. Both these marine organisms are excellent sources of **PS**, most of them sulphated (**sPS**). Although some similarities may be found between the **PS** from each group of organisms, they can be very heterogeneous and structurally different. The biological source and biodegradability of these biopolymers, coupled to the large variety of functionalities they encompass, make them promising compounds for the application in pharmaceuticals, therapeutics, and regenerative medicine. Some of the beneficial bioactivities demonstrated by the crude **PS** and their derivatives, either *in vitro* or *in vivo*, include anticoagulant and/or antithrombotic properties, immunomodulatory ability, antitumor and cancer preventive activity. They are also good antilipidaemic and hypoglycaemic agents, and can be powerful antioxidants, antibiotics and anti-inflammatory. Other biomedical properties of **PS** have been discussed, such as antinociceptive, angiogenic, cardioprotective, gastroprotective and hepatoprotective activities. The biomedical applications and potentialities of **PS** in this area were listed, such as healing wound agents, mucobioadhesives for bone and soft tissue closure, biolubricants to mitigate joint disorders caused by arthritis, vaccines for cancer immunotherapy, or in a new generation of biotextiles and medical fibres, in drug delivery systems, and scaffolds in regenerative medicine.

From the extensive list above, the importance of this type of compounds—**PS** from macro- and microalgae—for medical use is quite obvious. However, despite all the interesting properties and potentialities for human health, the use of these **PS**, especially those from microalgae need to be further explored. In particular, the toxicity and bioavailability of some of these polymers are yet to be studied on humans.
